# Identification of target genes regulated by the *Drosophila* histone methyltransferase Eggless reveals a role of Decapentaplegic in apoptotic signaling

**DOI:** 10.1038/s41598-018-25483-9

**Published:** 2018-05-08

**Authors:** Igojo Kang, Yourim Choi, Sueun Jung, Jae Yun Lim, Dooyoung Lee, Sumeet Gupta, Woongjoon Moon, Chanseok Shin

**Affiliations:** 1 Department of Agricultural Biotechnology, Seoul National University, Seoul, 08826 Republic of Korea; 2 Whitehead Institute for Biomedical Research, Cambridge, 02142 MA USA; 3 Research Institute of Agriculture and Life Sciences, and Plant Genomics and Breeding Institute, Seoul National University, Seoul, 08826 Republic of Korea

## Abstract

Epigenetic gene regulation is essential for developmental processes. Eggless (Egg), the *Drosophila* orthologue of the mammalian histone methyltransferase, SETDB1, is known to be involved in the survival and differentiation of germline stem cells and piRNA cluster transcription during *Drosophila* oogenesis; however the detailed mechanisms remain to be determined. Here, using high-throughput RNA sequencing, we investigated target genes regulated by Egg in an unbiased manner. We show that Egg plays diverse roles in particular piRNA pathway gene expression, some long non-coding RNA expression, apoptosis-related gene regulation, and Decapentaplegic (Dpp) signaling during *Drosophila* oogenesis. Furthermore, using genetic and cell biological approaches, we demonstrate that ectopic upregulation of *dpp* caused by loss of Egg in the germarium can trigger apoptotic cell death through activation of two pro-apoptotic genes, *reaper* and *head involution defective*. We propose a model in which Egg regulates germ cell differentiation and apoptosis through canonical and noncanonical Dpp pathways in *Drosophila* oogenesis.

## Introduction

Epigenetic regulation is a fundamental mechanism required for proper gene expression, cell proliferation, and survival during development^1^. Histone modifications, such as methylation and acetylation, play important roles in epigenetic regulation through global transcriptional changes^2^. For example, Histone 3 methylated at lysine9 (H3K9me) recruits Heterochromatin Protein 1a (HP1a), to establish the formation of heterochromatin^3,4^. Thus, H3K9me was generally considered to be involved in transcriptional repression^5^ but it was also reported that H3K9me was associated with transcriptionally active genes^6^, suggesting that the effects of H3K9me may be largely context-dependent.

Histone methyltransferases play critical roles in developmental processes at the epigenetic level and are known to be involved in a diverse range of diseases^2^. In *Drosophila* there are three different H3K9 methyltransferases, Su(var)3–9, G9a and Eggless (Egg, also known as dSETDB1). Among these, Egg, the *Drosophila* orthologue of the mammalian histone methyltransferase SETDB1, is the only H3K9 methyltransferase essential for viability and fertility^7–10^. Strong *egg* mutants show reduced levels of histone 3 lysine 9 trimethylation (H3K9me3) and concomitant oogenesis defects at the earliest stages in the germarium^7,10^. More specifically, *egg* mutant ovaries show defects in the survival of germline cells and surrounding somatic cells at very early stages of *Drosophila* oogenesis, resulting in rudimentary ovaries associated with apoptosis^7,10^. More recently, mosaic analyses revealed that Egg is required intrinsically for maintenance of both germline stem cells (GSCs) and follicle stem cells (FSCs)^11^. The same study also showed that Egg plays not only cell-autonomous but also non-cell-autonomous roles in somatic cells that affect the early differentiation of GSCs in the germarium^11^.

An interesting insight into the link between Egg function and P-element-induced wimpy testis (Piwi)-interacting small RNA (piRNA) production has also been proposed^12^. Rangan *et al*.^12^ showed that Egg is required to regulate piRNA cluster transcription through the deposition of H3K9me3, and for the repression of transposable elements (TEs). TEs are DNA sequences that can change their location in the genome and it has therefore been considered that TEs can generate DNA damage and genomic instability^13^. Meanwhile, piRNAs are mainly derived from TEs and other repeated elements and can silence target TEs to preserve genome integrity in animal germ cells^14^. Given the increased occurrence of apoptosis at early stages of oogenesis in *egg* mutant germline and somatic cells^7,10^,^11^, activation of TEs by a reduction of piRNAs in *egg* mutants may result in an increase in DNA damage, which in turn may trigger cell death. However, direct evidence is required to clarify whether the activation of TEs in *egg* mutants is indeed sufficient to cause DNA damage-induced apoptosis.

Despite the above-mentioned findings, the underlying mechanisms behind these observations remain to be determined. In particular, identification of target genes regulated by Egg may be crucial. To better understand the role of Egg in oogenesis, we adopted high-throughput RNA sequencing (RNA-seq). It has been shown that RNA-seq linked to bioinformatic analysis is a powerful tool to reveal unknown details of transcriptomic and epigenomic changes in an unbiased manner^15–17^. In the present study, we used RNA-seq to investigate *egg* mutation-induced transcriptional changes that may well be missed by conventional methods, thereby uncovering unprecedented insights into the role of Egg in oogenesis.

## Results

**Figure 1:**
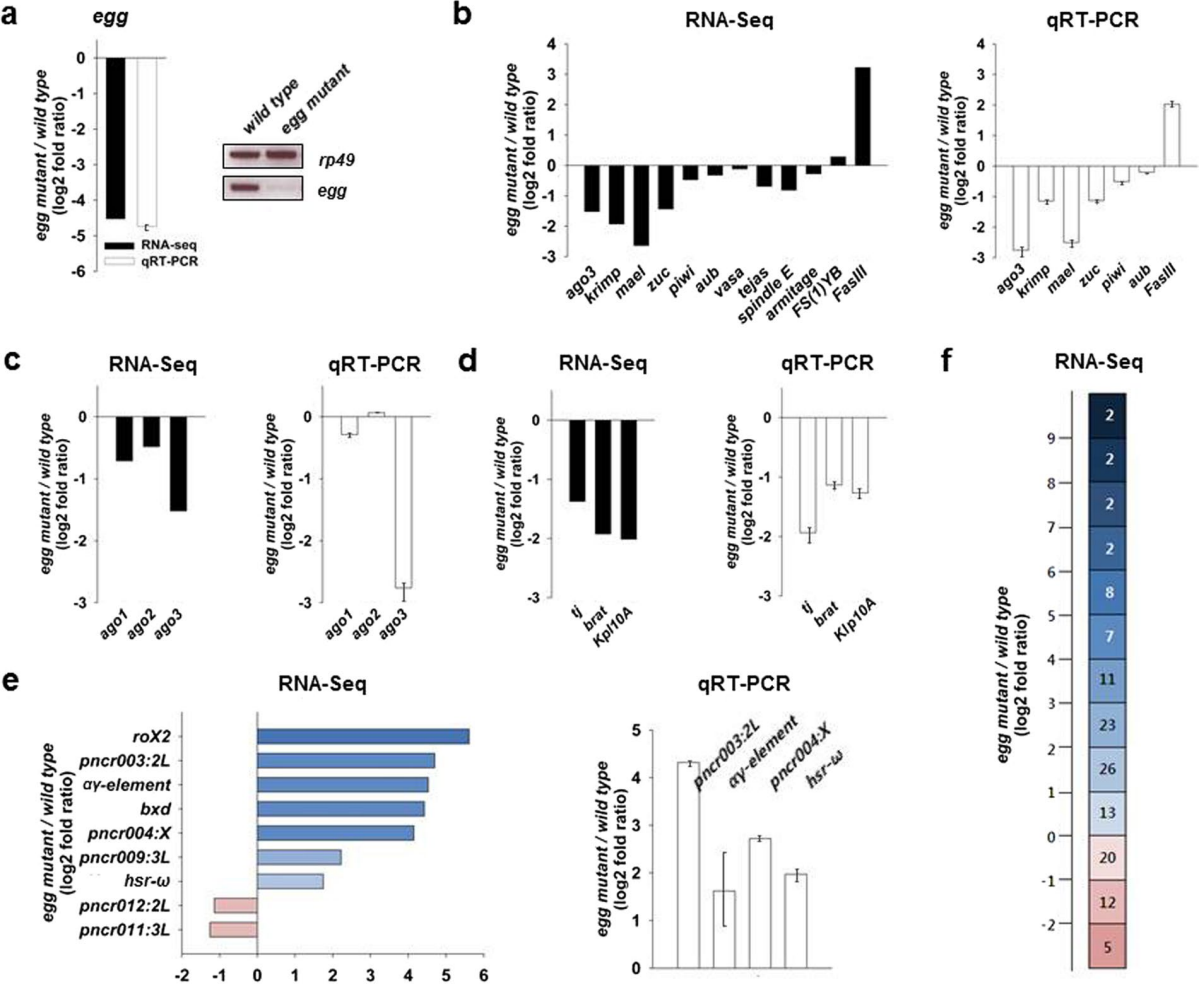
Egg regulates the expression of particular piRNA pathway genes and some lncRNAs. Differentially expressed genes by loss of Egg were identified from RNA-seq data (black bar) using the criteria of fold change ≥2. Fold changes are shown on the log_2_ scale (*egg* mutant/wild-type). qRT-PCR assays (white bar) were performed at least three times to confirm the RNA-seq data. Error bars represent standard deviation. (**a**) Expression level change of *egg* gene. *egg* expression was significantly reduced in *egg* mutant (*egg*^*2138*^*/Df(2R)Dll-Mp*) ovaries compared with wild-type ovaries. *rp49* was used as a control. (**b**) Representative expression profiles of piRNA pathway genes. Expression levels of *ago3*, *krimp*, *mael*, and *zuc* were downregulated in *egg* mutant ovaries. Consistent with *zuc* downregulation, *FasIII* expression was upregulated in *egg* mutant ovaries. (**c**) Expression level changes of Ago family members. Unlike *ago3*, expression of *ago1* and *ago2* was not significantly changed in *egg* mutant ovaries. (**d**) Expression level changes of genes for genic piRNAs. Expression levels of *tj*, *brat*, and *klp10A* genes which contain 3′-UTR piRNA clusters were downregulated in *egg* mutant ovaries. (**e**) Representative expression profiles of lncRNAs. Some well-known *Drosophila* lncRNAs, such as *roX2*, *αγ-element*, *bxd*, and *hsr-ω* were upregulated whereas some putative lncRNA genes, such as *pncr011:3L* and *pncr012:2L*, were downregulated in *egg* mutant ovaries. (**f**) Summary of expression profiles of putative lncRNA genes. Differentially expressed putative lncRNA genes are shown with the number of genes. The fold change on the log_2_ scale is placed on the vertical axis. Eighty-three genes were upregulated (≥ + 1) whereas 17 genes were downregulated (−1≥) in *egg* mutant ovaries. See also Supplementary Table S2.

To examine the gene expression changes caused by loss of Egg, we produced RNA-seq data from the anterior portion of wild-type (*w*^*1118*^) ovaries and heterozygous *egg* mutant (*egg*^*2138*^*/Df(2R)Dll-Mp*) ovaries carrying an early premature stop codon (*egg*^*2138*^) and a deficiency for the gene region, *Df(2R)Dll-Mp*^11^. As an internal control, we initially examined the change in *egg* gene expression (Fig. 1a). The expression of *egg* gene was strongly reduced in *egg* mutant ovaries compared with wild-type ovaries, which was further confirmed by quantitative Real Time-PCRs (qRT-PCR) (Fig. 1a) as well as Western blots (Supplementary Fig. S2). A total of 2627 upregulated genes and 1897 downregulated genes resulting from loss of Egg were identified from the RNA-seq data using DESeq with the criteria: normalized value ≥ 100 in either sample, and fold change ≥ 2. Genes were examined for enrichments in Gene Ontology biological processes^18^. The full gene list is available in the Gene Expression Omnibus (GEO) database (see Methods). To confirm the RNA-seq data, we also performed validation tests by qRT-PCR using separate RNA obtained from wild-type and *egg* mutant ovaries (the gene specific primers used for qRT-PCR are listed in Supplementary Table S1).

### Egg regulates the expression of particular piRNA pathway genes

Egg is required for transcription of all major piRNA clusters and for repression of TEs^12^. To better understand the underlying mechanism of Egg-associated piRNA production, we examined whether loss of Egg affects the expression levels of any known genes involved in the piRNA pathway by analyzing the RNA-seq data. Our results showed that *argonaute-3* (*ago3*), *krimper* (*krimp*), *maelstrom* (*mael*), and *zucchini* (*zuc*) were downregulated, but that expression levels of *piwi*, *aubergine (aub)*, *vasa*, *tejas*, *spindle E*, *armitage*, and *FS(1)YB* genes were not significantly changed by loss of Egg (Fig. 1b). Consistently, expression levels of Ago3, Krimp, and Mael proteins appeared reduced in *egg* mutant ovaries (Supplementary Figs S1 and S2). Zuc has been suggested as a key player in *traffic jam* (*tj*)-derived piRNA production, and FasIII is considered as a primary target of the *tj*-derived piRNA-Piwi complex^19^. Consistent with our finding of *zuc* downregulation in *egg* mutant ovaries, *FasIII* was upregulated in *egg* mutant ovaries (Fig. 1b). The *Drosophila* Ago family comprises five members: Ago1, Ago2, Ago3, Piwi, and Aub. Among these five members, the Piwi protein subfamily (Piwi, Ago3, and Aub) is essential for the production of piRNAs and transposon silencing to protect germline cells^20^. In *Drosophila* ovaries, piRNAs bind to the Piwi subfamily proteins to form the piRNA-induced silencing complex (piRISC). Meanwhile, Ago1 is mainly involved in repressing the translation of mRNAs by binding to microRNAs (miRNAs) to form the miRISC, and Ago2 associates with small interfering RNAs (siRNAs) to form the siRISC^21^. Because *ago3* expression was downregulated but the expression levels of *piwi* and *aub* were not significantly changed in *egg* mutant ovaries, we also examined expression level changes of the other *Drosophila* ago family members, Ago1 and Ago2. We found that the expression levels of *ago1* and *ago2* genes were not significantly changed in *egg* mutant ovaries (Fig. 1c). In addition to major piRNA clusters, such as the 42AB and flamenco clusters, the 3′-UTRs of *tj*, *brain tumor* (*brat*), and *klp10A* (a kinesin-13 of *Drosophila*) genes were also determined as new piRNA clusters that produce somatic piRNAs^19^. The role of Egg in these new piRNA clusters has not been investigated; therefore, we examined expression level changes of these genes in *egg* mutant ovaries. Consistent with the previously suggested notion that Egg plays a positive role in major piRNA cluster transcription^12^, the expression levels of *tj*, *brat*, and *klp10A* genes were all downregulated in *egg* mutant ovaries (Fig. 1d). Taken together, our results demonstrate that Egg not only promotes piRNA cluster transcription, but also plays a positive role in the expression of particular components of the piRNA machinery.

### Egg is involved in regulating expression of some long non-coding RNAs (lncRNAs)

On analysis of the RNA-seq data, we noticed that some well-known *Drosophila* lncRNAs were upregulated in *egg* mutant ovaries, prompting us to investigate whether Egg is involved in the regulation of lncRNA expression. In general, lncRNAs are transcripts longer than 200 nucleotides with little or no protein-coding capacity^22^. In addition to small non-coding RNAs such as piRNAs, miRNAs, and siRNAs, accumulating evidence suggest that lncRNAs also play key regulatory roles in diverse biological processes^22^.

We found that the expression levels of about 100 potential lncRNAs were significantly changed in *egg* mutant ovaries. A selected subset of well-known differentially expressed *Drosophila* lncRNAs is shown in Fig. 1e (the entire list is in Supplementary Table S2). Most of them (83%), including *roX2*, *αγ-element*, *bxd*, *hsr-ω*, *pncr003:2L*, *pncr004:X*, and *pncr009:3L*, were upregulated, whereas 17% of them, including *pncr011:3L* and *pncr012:2L*, were downregulated by loss of Egg (Fig. 1f). *Drosophila pncr* (*putative noncoding RNA*) genes are a set of putative lncRNA genes identified by positional curation^23^. Overall, our data suggest that Egg is involved in regulating expression of some lncRNA genes and that Egg generally plays a repressive role in the expression of these lncRNA genes.

### Two pro-apoptotic genes, *reaper* (*rpr*) and *head involution defective* (*hid*), are derepressed in *egg* mutant ovaries

**Figure 2:**
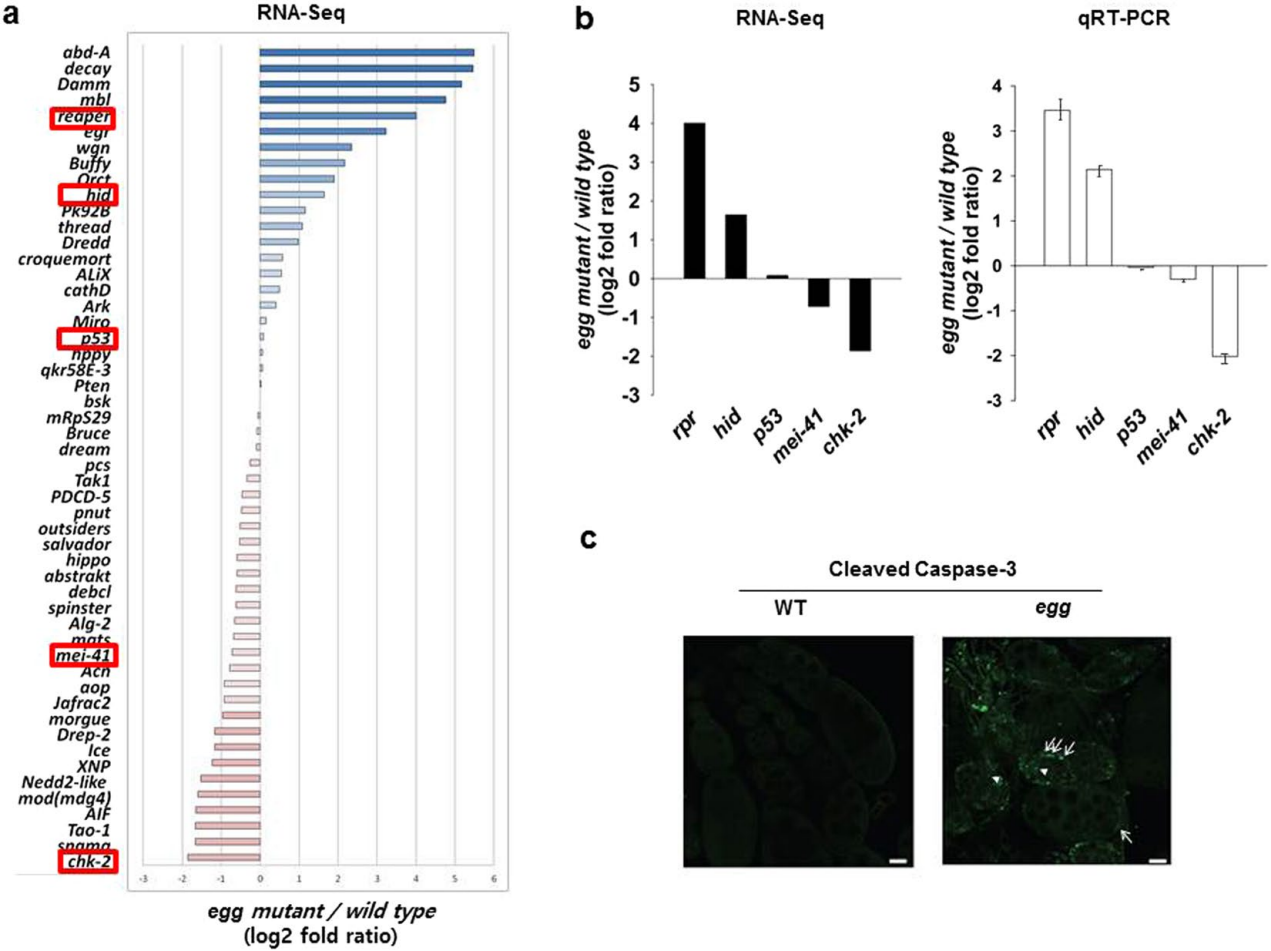
Apoptotic genes are generally derepressed in *egg* mutant ovaries. (**a**) Transcriptome analysis of apoptosis related genes using RNA-seq data. Overall, expression of apoptotic genes appears derepressed in *egg* mutant ovaries compared with wild-type ovaries. Fold changes are shown on the log_2_ scale (*egg* mutant/wild-type). Red boxes indicate representative apoptotic genes further analyzed by qRT-PCR. (**b**) RNA-seq (black bar) and qRT-PCR (white bar) analyses of the expression of five representative genes (*rpr*, *hid*, *p53*, *mei-41*, and *chk-2*). Expression levels of *rpr* and *hid* were upregulated whereas *chk-2* expression was downregulated in *egg* mutant ovaries compared with wild-type ovaries. Expression levels of *p53* and *mei-41* were not significantly changed. Fold changes are shown on the log_2_ scale (*egg* mutant/wild-type). qRT-PCR assays were performed at least three times. Error bars represent standard deviation. (**c**) Ovaries labeled with anti-cleaved Caspase-3 antibody. No apoptotic cells were observed in wild-type ovaries (left panel). Apoptotic germline (arrowhead) and somatic (arrow) cells were detected in *egg* mutant ovaries (right panel). Scale bars represent 20 µm.

Loss of piRNAs in germ cells leads to activation of TEs and consequently to an increase in DNA damage, which in turn can trigger apoptotic cell death^13,14^. Indeed, *egg* mutant females show rudimentary ovaries, resulting from apoptosis in the germaria at early stages of oogenesis^7,10,11^. We confirmed whether apoptotic events indeed happened in *egg* mutant ovaries using cleaved Caspase-3 antibody. As shown in Fig. 2c, we observed apoptotic germline and somatic cells in *egg* mutant ovaries, but not in wild-type ovaries. Thus, we compared expression level changes of apoptosis-related genes between wild-type and *egg* mutant ovaries using the RNA-seq data. As shown in Fig. 2a, the overall expression of apoptotic genes was seemingly derepressed in *egg* mutant ovaries. In particular, two pro-apoptotic genes, *rpr* and *hid* were upregulated in *egg* mutant ovaries (Fig. 2b). In the case of *grim*, the normalized value was too low in both wild-type and *egg* mutant ovaries to consider statistically significant. However, the expression levels of *p53* and *meiotic 41* (*mei-41/ATR*, the *Drosophila* ATM/ATR homologue) genes were not significantly changed and the expression level of the *maternal nuclear kinase* (*mnk/chk-2*, the *Drosophila* Chk-2 homologue) was downregulated in *egg* mutant ovaries (Fig. 2b). In *Drosophila*, it is well established that three pro-apoptotic genes, *rpr*, *hid*, and *grim* play key roles in the activation of apoptotic cell death, and each of the three pro-apoptotic genes is sufficient to induce apoptotic cell death in a Caspase-dependent manner^24^: During apoptosis, Rpr, Hid, and Grim proteins bind to the caspase inhibitor Diap1, thereby releasing activated caspases^24^. It has been known that most developmental apoptosis in *Drosophila* is initiated by the activation of the *rpr*, *hid*, and *grim* genes, and the protein activity of these genes is controlled mainly at the transcriptional level^24^.

Previously, immunostaining analyses revealed that Bag of marbles (Bam)-negative undifferentiated (i.e. GSC-like) cells were accumulated in *egg* mutant germaria, suggesting that Egg is required for Bam-dependent cystoblast differentiation^12,25^. However, *egg* mutant females show small ovaries, suggesting that the overall size of Bam-negative cells in *egg* mutant ovaries does not increase, probably because of apoptotic cell death in *egg* mutant germaria^7,10,11^. Our data suggest that the upregulation of *rpr* and *hid* genes in *egg* mutant ovaries may, at least partly, account for the previously observed apoptotic cell death in *egg* mutant germaria^7,10,11^.

### Expression levels of *decapentaplegic* (*dpp*) and *division abnormally delayed* (*dally*) are upregulated in *egg* mutant ovaries

In the *Drosophila* ovary, two or three GSCs reside in the GSC niche at the anterior tip of the germarium^26^. Following GSC division, one daughter cell moves out of the niche and changes its identity to a differentiating cystoblast by upregulating Bam, a key differentiation factor^26^. Maintenance of GSC identity depends on Dpp, the *Drosophila* orthologue of Bone morphogenetic protein 2/4 (BMP2/4), together with Dally, a cell-surface glypican that enhances Dpp signaling^27^. Thus, Dpp should be strictly regulated. In the GSC niche, somatic cap cells produce the majority of the Dpp signal whereas escort cells (ECs), which lie close to the cap cells, do not express the Dpp signal^27^. Dpp acts through the canonical signal transduction pathway involving the type I receptors Thickveins (Tkv) and Saxophone (Sax), and the type II receptor Punt^27^. In GSCs, Dpp signaling transcriptionally represses *bam*, which results in derepression of the essential downstream translational repressor Nanos (Nos)^28^. Meanwhile, in cystoblasts, *bam* expression is derepressed and Bam inhibits Nos/Pumilio (Pum) activity to initiate the differentiation process^28^.

**Figure 3:**
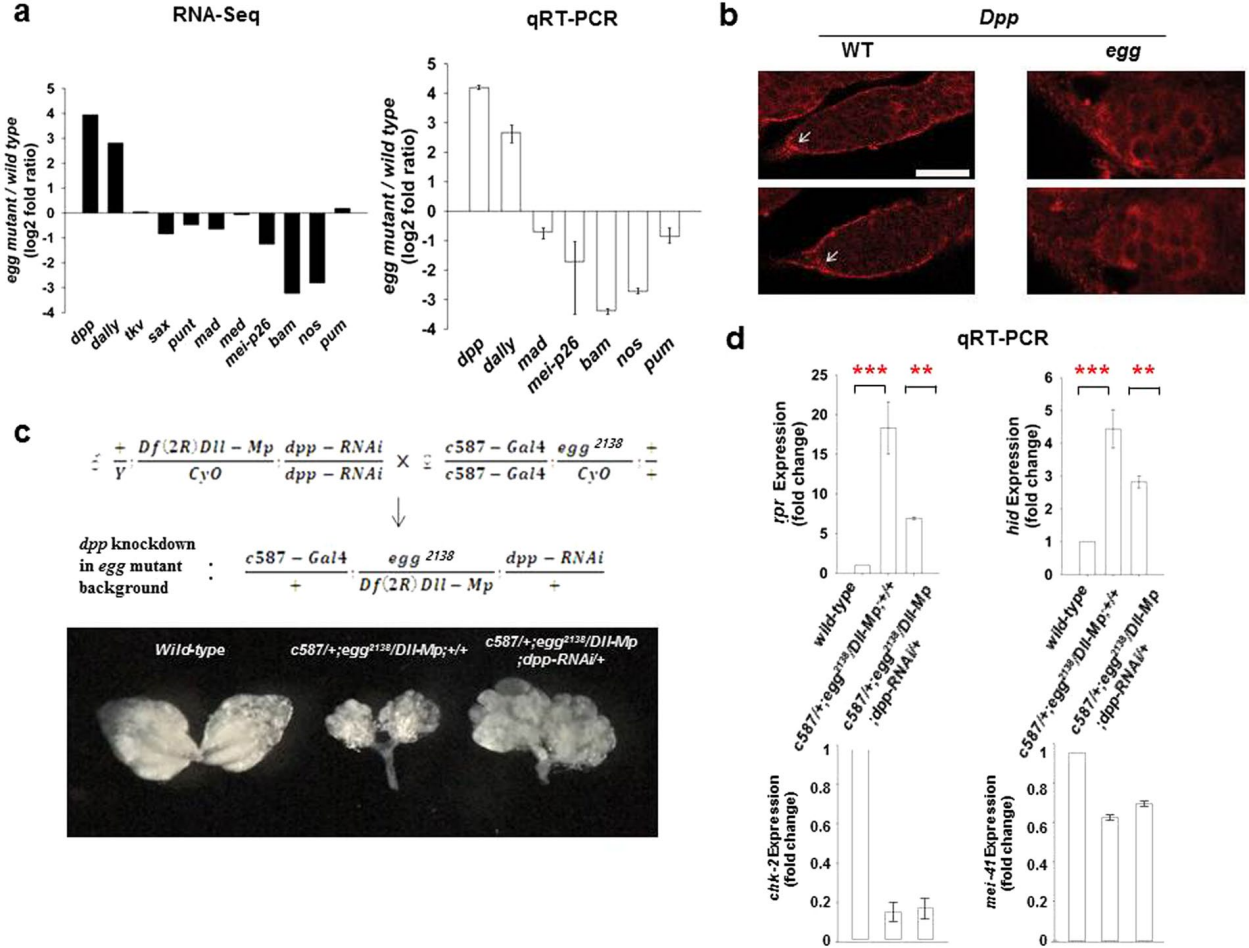
RNAi-mediated *dpp* knockdown in *egg* mutant germarium causes an increase in ovary size. (**a**) Representative expression profiles of genes involved in Dpp signaling. RNA-seq data analysis (black bar) revealed that expression levels of *dpp* and *dally* were upregulated whereas those of *nos* and *mei-P26* were downregulated in *egg* mutant ovaries compared with wild-type ovaries. Consistent with *dpp* and *dally* upregulation, *bam* was downregulated. Expression levels of *tkv*, *sax*, *punt*, *mad*, *med*, and *pum* were not significantly changed. Fold changes are shown on the log_2_ scale (*egg* mutant/wild-type). qRT-PCR assays (white bar) of the expression of seven representative genes (*dpp*, *dally*, *mad*, *mei-P26*, *bam*, *nos*, and *pum*) were performed at least three times. Fold changes are shown on the log_2_ scale (*egg* mutant/wild-type). Error bars represent standard deviation. (**b**) Germaria stained with anti-Dpp antibody. Dpp was detected specifically at the anterior tip (arrow) of wild-type germaria (left panels). Dpp appears increased and diffused from the anterior tip of *egg* mutant germaria (right panels). Scale bar represents 20 µm. (**c**) Representative light microscopic image of ovaries. RNAi-mediated *dpp* knockdown in *egg* mutant germarium causes an increase in ovary size. Ovaries of *egg* mutants (middle) are severely reduced in size compared with wild-type ovaries (left). 15–20% (n = 630) of *egg* mutant ovaries also carrying *c587-Gal4* and *dpp-RNAi* transgenes (*c587-Gal4*/+*; egg*^*2138*^*/Df(2R)Dll-Mp; dpp-RNAi*/+, right) showed an increase in size compared to *egg* mutant ovaries (*c587-Gal4*/+*; egg*^*2138*^*/Df(2R)Dll-Mp;* +/+, middle). (**d**) qRT-PCR assays. The increased expression levels of *rpr* and *hid* in *egg* mutant ovaries (*c587-Gal4*/+*; egg*^*2138*^*/Df(2R)Dll-Mp;* +/+) were significantly reduced in *egg* mutant ovaries also carrying *c587-Gal4* and *dpp-RNAi* transgenes (*c587-Gal4*/+*; egg*^2138^*/Df(2R)Dll-Mp; dpp-RNAi*/+) (one-way ANOVA with Bonferroni; **P < 0.01, ***P < 0.001; error bars represent standard deviation of three biological replicate experiments). RNAi-mediated *dpp* knockdown in *egg* mutant germarium did not change the expression levels of *chk-2* and *mei-41* in *egg* mutant ovaries.

We investigated whether loss of Egg leads to transcriptional changes of the genes involved in Dpp signaling. Consistent with previous reports^12,25^, our data show that *bam* was downregulated in *egg* mutant ovaries (Fig. 3a), which was further confirmed by immunostaining of ovaries with anti-Bam (Supplementary Fig. S1). Moreover, we found that *dpp* and *dally* were upregulated, but the expression levels of *tkv*, *sax*, *punt*, *mother against dpp* (*mad*), and *medea* (*med*) were not significantly changed in *egg* mutant ovaries (Fig. 3a), suggesting that the increased expression levels of *dpp* and *dally* genes by loss of Egg may be responsible for Bam-dependent cystoblast differentiation defects in *egg* mutant germaria^12,25^. Consistently, we detected a significantly increased expression level of Dpp protein in *egg* mutant ovaries by Western blots (Supplementary Fig. S2) and immunostaining (Fig. 3b): We detected quite increased and diffused signals of Dpp protein from the anterior tip of *egg* mutant germaria compared with a specific signal of Dpp protein at the anterior tip of wild-type germaria (Fig. 3b). Notably, however, we also found that *nos* was downregulated in *egg* mutant ovaries (Fig. 3a). These results suggest that some of the Bam-negative cells in *egg* mutant germaria^12,25^ may also be Nos-negative cells, or the typical identity of the accumulated germ cells in *egg* mutant germaria^12,25^ may not be simply Bam-negative but also Nos-negative (i.e. GSC-like + cystoblast-like). Indeed, consistent with our results, germline clone analysis showed that *egg*-negative germline cell clones developed normally up to stage 5 of oogenesis but died through apoptosis^11^.

### RNAi-mediated *dpp* knockdown in *egg* mutant germarium causes an increase in ovary size

To evaluate the significance of *dpp* upregulation to *egg* mutant ovary phenotypes, we knocked down *dpp* expression using RNAi in *egg* mutant background. We first tested the effect of the previously tested *dpp-RNAi* transgene^29^ in *egg* mutant background under the control of the *actin5C-Gal4* driver, a highly and ubiquitously active driver. However, as expression of the *dpp-RNAi* transgene by the *actin5C-Gal4* driver resulted in pupal lethality, we could not evaluate the effect of *dpp* knockdown by the *act5C-Gal4* driver on *egg* mutant ovaries. Given previous results that RNAi-mediated reduction of *dpp* expression by the *c587-Gal4* driver, a driver active in ECs and early follicle cells, suppressed the ovarian tumor phenotype induced by *H3K4 demethylase 1-RNAi*^29^, we next evaluated the effect of *dpp* knockdown in *egg* mutant background using the *dpp-RNAi* transgene and the *c587-Gal4* driver. We crossed flies carrying the *dpp-RNAi* transgene and *Df(2R)Dll-Mp* deficiency, which uncovers the *egg* gene, (+/*Y; Df(2R)Dll-Mp/CyO; dpp-RNAi/dpp-RNAi*) with flies carrying the *egg* mutation and *c587-Gal4*. (*c587-Gal4/c587-Gal4; egg*^*2138*^*/CyO*; +/+). We found that a small but consistent percentage (17.33 ± 1.81%, n = 630) of the *egg* mutant ovaries also carrying *c587-Gal4* and *dpp-RNAi* transgenes (*c587-Gal4*/+*; egg*^*2138*^*/Df(2R)Dll-Mp; dpp-RNAi*/+) showed an increase in size (Fig. 3c). Ovaries of the genotype (*c587-Gal4*/+*; egg*^*2138*^*/Df(2R)Dll-Mp; dpp-RNAi*/+) contained an accumulation of disorganized and somewhat bloated ovarioles and exhibited differentiation defects (data not shown) similar to those described previously^12,25^, raising the possibility that, unlike *egg* mutant germline and surrounding somatic cells (*c587-Gal4*/+*; egg*^*2138*^*/Df(2R)Dll-Mp*; +/+), the defective cells in the *dpp*-knockdown + *egg* mutant ovaries (*c587-Gal4*/+*; egg*^*2138*^*/Df(2R)Dll-Mp; dpp-RNAi*/+) could be maintained for a longer time by escaping apoptosis. Indeed, our qRT-PCR results showed that the increased expression levels of *rpr* and *hid* genes in *egg* mutant ovaries (*c587-Gal4*/+*; egg*^*2138*^*/Df(2R)Dll-Mp;* +/+) were significantly reduced in the *dpp*-knockdown + *egg* mutant ovaries (*c587-Gal4*/+*; egg*^*2138*^*/Df(2R)Dll-Mp; dpp-RNAi*/+) (Fig. 3d).

### *dpp* overexpression in *Drosophila* ovarian somatic cells (OSCs) induces cell death and activates the expression of *rpr* and *hid*

**Figure 4:**
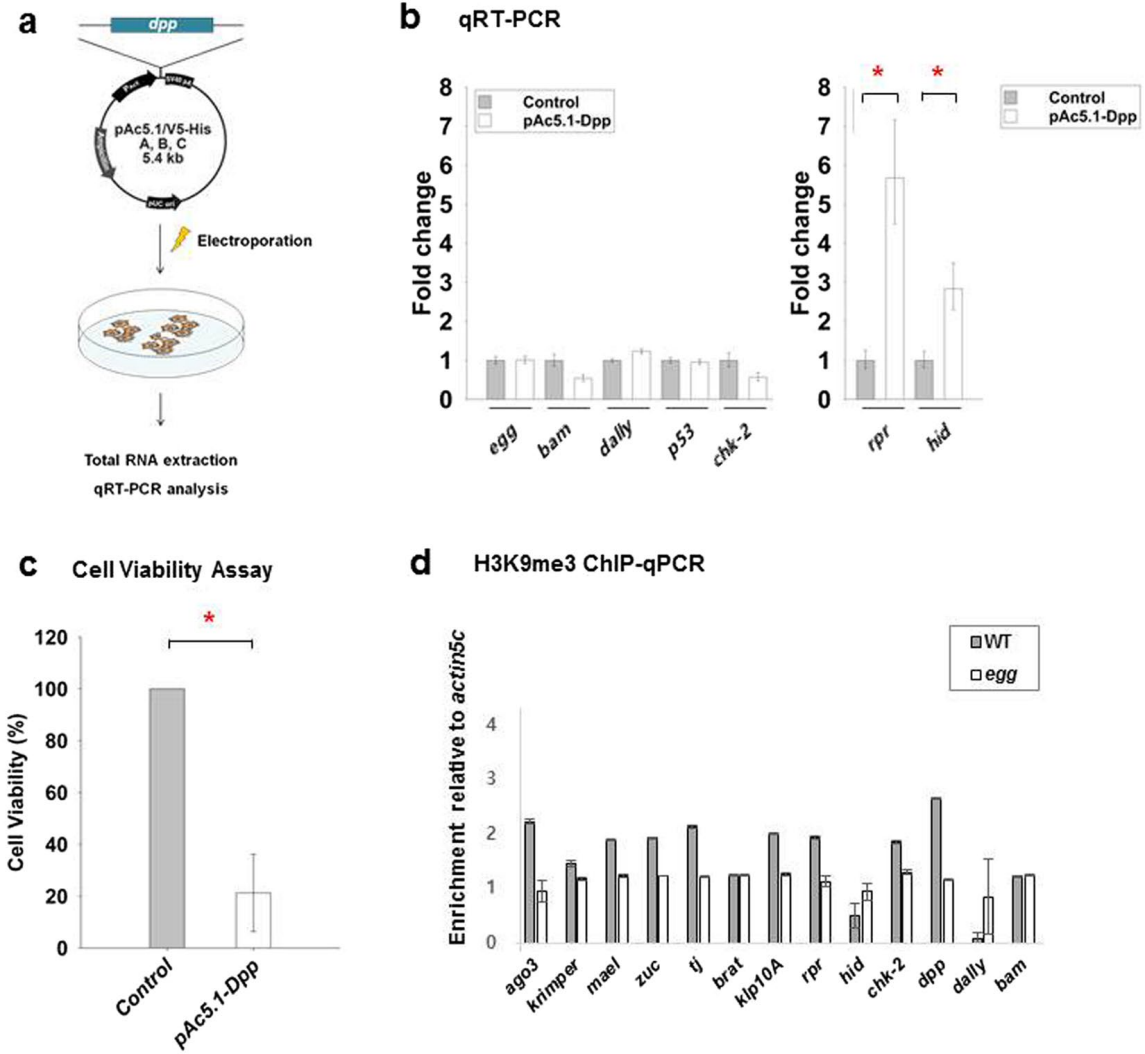
*dpp* overexpression induces apoptotic cell death through activation of *rpr* and *hid* in *Drosophila* ovarian somatic cells (OSCs). (**a**) Schematic illustration of Dpp overexpression experiments in OSCs. (**b**) qRT-PCR assays. *dpp* overexpression activated the expression of *rpr* and *hid* in OSCs. qRT-PCR assays show changes in expression levels of seven representative genes (*egg*, *bam*, *dally*, *p53*, *chk-2*, *rpr*, and *hid*) following *dpp* overexpression in OSCs (unpaired *t*-test; *P < 0.05; error bars represent standard deviation of three biological replicate experiments). (**c**) MTS assays. *dpp* overexpression induced cell death in OSCs. MTS assays show that the viability of *dpp*-overexpressing OSCs is significantly decreased (unpaired *t*-test; *P < 0.05; error bars represent standard deviation of three biological replicate experiments). (**d**) Chromatin immunoprecipitation (ChIP)-qPCR assays. ChIP was performed using H3K9me3 antibody to measure enrichment levels of H3K9me3 in the putative target genes of Egg. The recovered DNA was analyzed by qPCRs. Certain levels of H3K9me3 were present in some of the putative target genes such as *dpp*, *rpr*, *chk-2*, *ago3*, *zuc*, *mael*, *tj*, and *klp-10A* genes. H3K9me3 levels in these genes were significantly reduced in *egg* mutant ovaries. The enrichment levels are relative to *act5C*. Error bars represent standard deviation of three biological replicate experiments.

To test whether *dpp* upregulation in ovarian somatic cells can induce cell death directly, we transfected a Dpp expression construct into OSCs^19^, and evaluated cell viability and changes in gene expression following *dpp* overexpression (Fig. 4a). *Drosophila* OSCs are composed of mitotically active early somatic cells derived from the parental female germline stem/ovarian somatic sheath (fGS/OSS) cell line^19^. Our qRT-PCR results showed that expression levels of *rpr* and *hid* were significantly elevated following *dpp* overexpression in OSCs (Fig. 4b). Consistent with the qRT-PCR results, MTS assays showed that *dpp* overexpression significantly decreased the viability of OSCs (Fig. 4c). Collectively, these data suggest that ectopic upregulation of *dpp* in ovarian somatic cells can induce cell death through activation of *rpr* and *hid*. We also examined the impact of loss of Egg on H3K9me3 enrichment levels in *dpp*, *dally*, *rpr*, and *hid* genes. Our results showed that certain levels of H3K9me3 were present in *dpp* and *rpr* genes, but not in *dally* and *hid* genes (Fig. 4d). Moreover, H3K9me3 levels in *dpp* and *rpr* genes were significantly reduced in the absence of Egg, suggesting that H3K9me3 could directly regulate the expression of *dpp* and *rpr* at least in part (Fig. 4d).

## Discussion

In the present study, we used RNA-seq data analysis and qRT-PCR validation to demonstrate that Egg plays diverse roles in the regulation of piRNA production, lncRNA expression, apoptosis-related gene expression, and Dpp signaling during *Drosophila* oogenesis. Furthermore, using genetic and cell biological approaches, we demonstrated that ectopic upregulation of *dpp* caused by loss of Egg in the germarium can trigger apoptosis *in vivo*.

Regarding piRNA production, our results revealed that among the known piRNA machinery components, *ago3*, *krimp*, *mael*, and *zuc* genes are the major piRNA-related targets of Egg (Fig. 1b). Consistent with the previously suggested role of Egg for promoting piRNA production, all of the putative target genes were downregulated in *egg* mutant ovaries (Fig. 1b,d). In *Drosophila*, two piRNA processing pathways, primary processing and secondary processing, have been proposed^14^; the primary processing pathway functions in both germline and somatic cells by processing precursor piRNAs into piRNAs whereas the secondary processing pathway functions only in germline cells in which piRNAs are amplified by the ping-pong cycle^14,30,31^. Zuc plays a central role in the primary processing pathway^19^. Given the involvement of Egg in both germline and somatic piRNA production^12^, we tested whether decreased expression of *zuc* is responsible for the ovarian phenotypes caused by loss of Egg. We investigated phenotypic changes after introduction of wild-type *zuc* transgenes^32^ under the control of the *actin5C-Gal4* driver into *egg* mutant background, but the ovaries of the genotypes (*egg*^*2138*^*/Df(2R)Dll-Mp; HA-tagged zuc* (or *EGFP-tagged zuc*)*/actin5C-Gal4*) did not show any significant phenotypic changes compared with those of *egg* mutants (data not shown). Ago3 is essentially involved in the secondary processing pathway along with Aub^14^. The nuage, which surrounds the nuclei of nurse cells, has been proposed as a site for the ping-pong cycle^30,31^. Various types of proteins, including Ago3, Krimp, and Mael, have been identified as nuage components^14^. The decreased expression levels of the particular nuage components in *egg* mutant ovaries (Fig. 1b and Supplementary Figs S1 and S2) suggest that the reduction of germline piRNAs in *egg* mutants may result from not only a reduction of precursor piRNA transcription, as proposed previously^12^, but also from a failure of the piRNA amplification pathway.

Our data also revealed a previously unknown role of Egg as an lncRNA regulator (Fig. 1e,f). In *Drosophila*, the existence of lncRNAs has long been known, but only a few lncRNAs have been investigated^33^. Here, we revealed that Egg is involved in regulating the expression of 100 potential lncRNA genes, and appears to play a repressive role in the expression of these lncRNAs (Fig. 1e,f). Among the upregulated lncRNAs in *egg* mutant ovaries, two well-known heat-inducible lncRNAs, *αγ-element* and *hsr-ω* are located in genomic regions where numerous TEs are found^34,35^. Given the involvement of Egg in regulating piRNA production and thus TE mobilization, this raises an intriguing possibility of a link between the upregulation of *αγ-element* and *hsr-ω* in *egg* mutant ovaries and their genomic locations as TE hotspots. A strong upregulation of *pncr003:2L* and *pncr004:X* by loss of Egg is noteworthy, but the functional significance of these lncRNAs in the *Drosophila* ovary has not been determined. Although we tried to knock down *pncr003:2L* and *pncr004:X* using transgenic flies containing dsRNA for RNAi of *pncr003:2L* or *pncr004:X* under the control of the *act5C-Gal4* driver, we could not detect any phenotypic changes during oogenesis. Interestingly, *pncr003:2L* was originally annotated as an lncRNA^23^, but it was recently reported to encode two small functional peptides that are involved in regulating calcium transport in the *Drosophila* heart^36^. Determination of the function of *pncr003:2L* in the *Drosophila* ovary is an interesting issue that needs to be addressed.

In this study, we demonstrated that *dpp* and *dally* were strongly upregulated in *egg* mutant ovaries (Fig. 3a) and that *dpp* knockdown in ECs and early follicle cells in *egg* mutant background resulted in an increase in the size of the ovaries (Fig. 3c). Previously, by analysis of *egg-RNAi* knockdown in ECs, an EC-specific requirement for *egg* in controlling germ cell differentiation was suggested^25^. Moreover, germ cell differentiation defects caused by *egg* knockdown in ECs was attributed to an increase in Dpp signaling because removal of one copy of *dpp* partially suppressed the tumorous phenotype caused by *egg* knockdown in ECs^25^. The expression level of *dally*, an enhancer of Dpp signaling, may be maintained at a high level in the *dpp* knockdown in *egg* mutant background (Fig. 3c); therefore, the enhancement of Dpp signaling caused by loss of Egg may be maintained in the *dpp* knockdown in *egg* mutant germarium, thereby exhibiting similar differentiation defects as those observed in the *egg* mutant germarium^12,25^. However, the defective cells in the *dpp*-knockdown in *egg* mutant background could be maintained for a longer time, which may be attributed to a reduction of the enhanced apoptosis in *egg* mutant ovaries because the increased expression levels of *rpr* and *hid* in *egg* mutant ovaries were significantly reduced in the *dpp*-knockdown in *egg* mutant background (Fig. 3d). Given that Egg is broadly expressed in germ cells and somatic cells in the ovary during oogenesis^7^ (Supplementary Fig. S1), *dpp* knockdown only in ECs and early follicle cells may not be sufficient to consistently counteract the overall apoptosis-promoting effect caused by *dpp* upregulation in *egg* mutant ovaries, which may explain the relatively low occurrence of the effect of the *dpp* knockdown in ECs and early follicle cells on the increase in ovary size.

We propose a model in which loss of Egg may initially cause a relatively mild level of ectopic *dpp* and *dally* overexpression that may be sufficient to cause germ cell differentiation defects through repression of *bam*. Apoptosis may then be initiated when the level of ectopic *dpp* overexpression reaches a certain threshold level capable of inducing *rpr* and *hid* upregulation perhaps through a *dpp* positive-feedback loop^37^. Alternatively, we cannot rule out the possibility that Egg represses *dpp*, *dally*, *rpr* and *hid* separately although the possibilities are not necessarily exclusive. Further studies are needed to determine the exact molecular mechanism by which Dpp signaling is increased in *egg* mutant ovaries. Given the role of BMPs in many mammalian stem cell systems^27^ and the existence of mammalian homologues of Egg and Dpp, the role of Egg in regulating Dpp signaling may provide important insights into their potential roles in mammalian stem cells.

## Methods

### *Drosophila* strains and genetics

All flies were maintained at room temperature (24–26 °C) on cornmeal-molasses medium. *w*^*1118*^ was used as a wild-type control. *egg*^*2138*^/*Df(2R)Dll-Mp* females were used for the isolation of ovarian tissues and phenotypic analysis. *egg*^*2138*^/*GFPCyO* and *Df(2R)Dll-Mp*/*GFPCyO* were provided by T. Hazelrigg (Columbia University, New York, NY, USA). *c587-Gal4* was provided by A. Spradling (Carnegie Institute for Science, Baltimore, MD, USA). Transgenic lines expressing N-terminally *triple-Hemaglutanin (HA)-tagged zuc* and *EGFP-tagged zuc* were provided by T. Schüpbach (Princeton University, Princeton, NJ, USA). *act5C-Gal4* and *dpp-RNAi* (BL-31531) lines were obtained from the Bloomington Stock Center. To knock down the expression of *dpp*, *c587-Gal4* and *dpp-RNAi* transgenes or *act5C-Gal4* and *dpp-RNAi* transgenes were introduced into the *egg*^*2138*^/*Df(2R)Dll-Mp* background by standard genetic crosses. To test the effect of wild-type *zuc* transgenes on the ovarian phenotype caused by loss of Egg, *HA-tagged zuc* and *act5C-Gal4* transgenes or *EGFP-tagged zuc* and *act5C-Gal4* transgenes were crossed into the *egg*^*2138*^/*Df(2R)Dll-Mp* background by standard genetic crosses. To knock down *pncr003:2L* or *pncr004:X*, transgenic flies containing dsRNA for RNAi of *pncr003:2L* (Bloomington Drosophila Stock Center, Stock Number 28957) or *pncr004:X* (Bloomington Drosophila Stock Center, Stock Number 28547) were used under the control of the *actin5C-Gal4* driver.

### RNA sequencing sample preparation and data analysis

Ovaries were dissected from 2- to 4-day-old *Drosophila* females and frozen in liquid nitrogen. *egg*^*2138*^*/Df(2R)Dll-Mp* females have rudimentary ovaries; therefore, mid- (stages 8–9) to late- (stages 10–14) stage wild-type egg chambers were removed manually to match the developmental stages and size. A representative image of egg chambers used for our RNA-seq and qPCRs is shown in Supplementary Fig. S1 as dashed lines. Total RNAs were extracted using TRIzol® reagent (Invitrogen). Isolated RNAs were treated with DNAse I (Takara) and further purified by acid phenol:chloroform extraction. cDNAs were synthesized from 1 μg of DNAse-treated total RNAs by reverse transcription (RT) with SuperScript III (Invitrogen). Twenty-microliter RT reactions containing 2.5 µM oligo- (dT)20 primers, 2.5 µM random hexamers, 0.5 mM each dNTP, 10X reverse transcription buffer, 5 mM MgCl_2_, 0.01 M DTT, 40 units/µl RNase-OUT™ and 200 units/µl SuperScript® III reverse transcriptase were incubated at 50 °C for 60 min, at 25 °C for 10 min and at 85 °C for 5 min. Raw sequences were aligned to the *Drosophila* genome (*Drosophila* genome version R5.42) using Burrows-Wheeler Aligner (BWA) software. Numbers of reads aligned within each gene were counted using the HTSeq package of Python and normalized with the DESeq package of R. Differentially expressed genes between *w*^*1118*^ and *egg*^*2138*^/*Df(2R)Dll-Mp* ovaries were identified using the DESeq criteria: normalized value ≥100 in either sample and fold change ≥2. All sequencing data has been deposited with the GEO database (Accession GSE87492).

### Quantitative Real-Time PCR

Relative mRNA levels were quantified using a LightCycler® 480 Real-Time PCR System (Roche). Each PCR reaction was carried out in 10 µl containing 5 µl LightCycler® 480 SYBR Green I Master (Roche), 0.25 µM gene specific primers and 20 ng cDNA. Reactions were run at 95 °C for 5 min followed by 45 cycles at 95 °C for 10 sec, 58 °C for 10 sec and 72 °C for 10 sec. Results were normalized to *ribosomal protein 49* (*rp49*). Gene expression levels were calculated using the comparative Ct method. All experiments were carried out at least three times for each of three biological replicates. The gene specific primers used for qRT-PCR are listed in Supplementary Table S1.

### Immunostaining

Ovaries were dissected in PBS, fixed for 9 min in a 6:1 mix of heptane:fixative (6% formaldehyde, 16.7 mM KPO_4_, 75 mM KCl, 25 mM NaCl, and 3.3 mM MgCl_2_), washed 3 × 5 min in PBS with 0.1% Triton-X100 (PBTX), and blocked for 4 hr in 1% BSA in PBTX. Primary and secondary antibodies were diluted in 1% BSA in PBTX. The ovaries were incubated overnight at 4 °C in primary antibody diluted in 1% BSA in PBTX, washed 3 × 10 min in PBTX, incubated for 3 hr at room temperature in secondary antibody (anti-rabbit Alexa Fluor 488 or anti-mouse Alexa Fluor 555, Abcam) at a dilution of 1:500, and washed 3 × 10 min in PBTX. Primary antibodies included rabbit anti-Cleaved Caspase-3 (1:500, Cell Signaling), mouse anti-Dpp (1:50, Santa Cruz Biotechnology), mouse anti-Bam-C (1:50, Developmental Studies Hybridoma Bank), mouse anti-Ago3 (1:250, H. Siomi), mouse anti-Krimp (1:250, H. Siomi), and rabbit anti-Egg (1:500). Anti-Egg polyclonal antiserum was raised against amino acids 79–315 of Egg in rabbits as described previously^7^. Leica SP8X confocal microscope was used to acquire images and images were processed with LAS X software.

### Chromatin immunoprecipitation

Approximately 300 ovary pairs of wild-type or *egg*^*2138*^*/Df(2R)Dll-Mp* females were used in each experiment. Chromatin preparation was carried out as described previously^38^. Sonication used Sonics Vibra-Cell VC130 Ultrasonic Processor at power 20, using 4 pulses of 10 sec with 50 sec intervals on ice. Immunoprecipitation was carried out using H3K9me3 antibody (3 µg, Millipore), then immunoprecipitated DNA fragments were quantified by qPCR. Enrichment of H3K9me3 is determined by normalization to a constitutively expressed *actin5C* gene or *egg* gene. The mean of the normalized value is reported using three biological replicates.

### Ovarian somatic cell (OSC) culture and overexpression of Dpp

The OSC line was a gift from H. Siomi (Keio University, Japan). Cells were grown at 26 °C in Shields and Sang M3 Insect Medium (Sigma) supplemented with 0.6 mg/ml glutathione, 10% FBS, 10 mU/ml insulin and 10% fly extract. A full-length *dpp* cDNA was PCR-amplified from *Drosophila* ovarian total cDNAs with gene specific primers. This *dpp* cDNA was tagged by the Flag epitope (MDYKDDDDK) at the N-terminus and was inserted between the EcoRI and XhoI sites of pAc5.1/V5-HisA (Invitrogen), to generate pAc5.1-Dpp, a Dpp expression vector. The primers used were as follows (5′-3′):

Forward: GAAGAATTCATGGACTACAAAGACGATGACGATAAAATGCGCGCATGGCTTCTACTCCT

Reverse: TTCCTCGAGCTATCGACAGCCACAGCCCACCAC

For Dpp overexpression in OSCs, 3 × 10^6^ trypsinized OSCs were resuspended in 100 μl of Solution V of the Cell Line Nucleofector Kit V (Amaxa Biosystems) and mixed with 5 μg of pAc5.1/V5-HisA or pAc5.1-Dpp. Transfection was conducted in an electroporation cuvette using a Nucleofector instrument (Amaxa Biosystems). The transfected cells were transferred to fresh OSC medium and incubated at 26 °C. After incubation for 48 hr, the cells were treated with TRIzol and total RNAs were extracted as described above.

### Cell viability assay (MTS assay)

Cell viability was measured using a CellTiter 96 Aqueous One Solution kit (Promega). 3 × 10^6^ trypsinized OSCs were transfected with 5 μg of pAc5.1/V5-HisA or pAc5.1-Dpp as described above. The transfected cells were seeded in 96-well plates and incubated at 26 °C. After incubation for 48 hr, the medium was changed to 10% M3/FBS medium. Then 20 µl of MTS solution was added to each well and cells were incubated at 26 °C for 3 hrs. Absorbance at 490 nm was measured using a Microplate Reader (Tecan). All experiments were carried out using three biological replicates.

## Electronic supplementary material

Supplementary Information
