# Supplementary material for: Identification of target genes regulated by the Drosophila histone methyltransferase Eggless reveals a role of Decapentaplegic in apoptotic signaling
Source: Sci Rep. 2018 May 8;8:7123. doi: 10.1038/s41598-018-25483-9 (PMC5940877; doi:10.1038/s41598-018-25483-9)
Supplement: Supplementary file 1 — Supplementary Information [file 41598_2018_25483_MOESM1_ESM.pdf]

Supplementary Information

Identification of target genes regulated by the *Drosophila* histone methyltransferase Eggless reveals a role of Decapentaplegic in apoptotic signaling

Igojo Kang[1†], Yourim Choi[1†], Sueun Jung[1], Jae Yun Lim[1], Dooyoung Lee[1], Sumeet Gupta[2], Woongjoon Moon[1]*, and Chanseok Shin[1,3]*

[1]Department of Agricultural Biotechnology, Seoul National University, Seoul, Republic of Korea; [2]Whitehead Institute for Biomedical Research, Cambridge, MA, USA; [3]Research Institute of Agriculture and Life Sciences, and Plant Genomics and Breeding Institute, Seoul National University, Seoul 08826, Republic of Korea

[†]these authors contributed equally to this work

*Corresponding authors:

Chanseok Shin, Department of Agricultural Biotechnology, Institute of Agriculture and Life Sciences, and Plant Genomics and Breeding Institute, Seoul National University, Seoul 08826, Republic of Korea. E-mail: cshin@snu.ac.kr

Woongjoon Moon, Department of Agricultural Biotechnology, Seoul National University, Seoul 08826, Republic of Korea. E-mail: wm9306@naver.com

Supplementary Figure S1. Immunostaining analysis of *egg* mutant ovaries

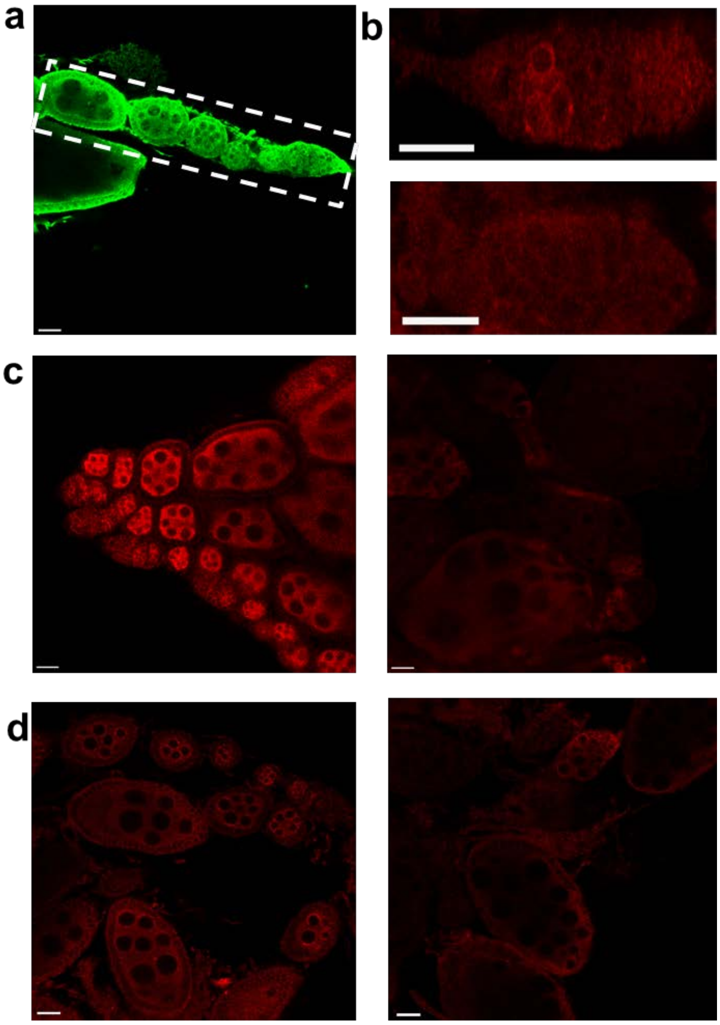

(a) A representative image of wild-type germarium and early-stage egg chambers used for RNA-seq and qPCR (dashed lines). The regions of the germarium and early-stage egg chambers are labeled with an antibody to Eggless. Eggless (green) is strongly detected in the regions of the germarium and early-stage egg chambers.

(b) Germaria labeled with an antibody to Bam (red). Bam is expressed in cystoblasts and early cystocytes in wild-type germarium (upper panel) but it is strongly reduced in *egg* mutant germarium (lower panel).

(c) Ovaries labeled with an antibody to Ago-3 (red). Expression level of Ago-3 protein appears reduced in *egg* mutant ovaries (right panel) compared with wild-type ovaries (left panel).

(d) Ovaries labeled with an antibody to Krimp (red). Expression levels of Krimp protein appears reduced in *egg* mutant ovaries (right panel) compared with wild-type ovaries (left panel).

Supplementary Figure S2. Western blot analysis of ovary extracts

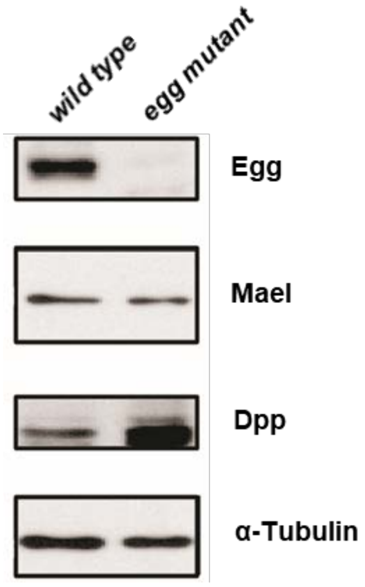

Western blots of ovary extracts labeled with antibodies to Egg, Mael, or Dpp. Egg protein is not detected in *egg* mutant (*egg*^{2138}*/Df(2R)Dll-Mp*) ovaries. An increase in expression level of Dpp protein is detected in *egg* mutant ovaries whereas Mael protein expression appears reduced in *egg* mutant ovaries.

Supplementary Table S1. Quantitative RT-PCR primers

Gene	Forward Primer	Reverse Primer
rp49	Forward 22mer<br>CCGCTTCAAGGGACAGTATCTG	Reverse 19mer<br>ATCTCGCCGCAGTAAACGC
eggless	Forward 20mer<br>GGCAACCTTGGACGCTATTT	Reverse 20mer<br>AACGCAGGTCGTGAGTATCC
ago1	Forward 20mer<br>GCGAGGTTTGGTTCGGTTTC	Reverse 20mer<br>CGTTGATGTCGCGAATGTCC
ago2	Forward 20mer<br>ACGCGTTTATCTGACTGGCA	Reverse 20mer<br>GACAATCGTTCGCTTTGCGT
ago3	Forward 20mer<br>CTGCATTTGTGGCCTCCATA	Reverse 20mer<br>GGGGAGTTTGCCATTCCTTT
krimp	Forward 20mer<br>TCGAAACCGCGTCTTCTTGA	Reverse 20mer<br>CCACGTTTTCACATGGAGCC
mael	Forward 20mer<br>AGACCACACTTCCTTTGCCG	Reverse 21mer<br>GTGCGTCGGTAAGTTCCTAGC
zuc	Forward 20mer<br>CCCCATTACCACGAACTTGA	Reverse 20mer<br>CCAAGAGCCGTCCAGTTTA
piwi	Forward 20mer<br>CAAGGCCGGATAATTGGACA	Reverse 20mer<br>CCATCGCTCGGAGTGGTAAG
aub	Forward 20mer<br>GGGATGACCAGCAAGAAAGG	Reverse 20mer<br>AAGACCGTCGCAGTCGTGTA
FasIII	Forward 20mer<br>AACCCAACACAGCGCTCCTC	Reverse 20mer<br>ATCCGGGTGTCTTGCTCCAC
tj	Forward 20mer<br>ACCAGTGGCACATGGACGAA	Reverse 20mer<br>CGCTCCCGAAGATGTGTTCA
brat	Forward 20mer<br>CGATTCGGCAATCTGCAGTG	Reverse 20mer<br>CGAAGACGAGGATGAGGACG
klp10A	Forward 20mer<br>ATGACCATGGCCGGAAACAT	Reverse 20mer<br>TCACGCTATTGCTGCTGACA
pncr003:2L	Forward 21mer<br>TTGTTCGGCAATGGAATCTAC	Reverse 22mer<br>GTACGAGTGCGAAATAAACGTG
αγ-element	Forward 20mer<br>CTTCGTCTACGGAGCGACAA	Reverse 22mer<br>GCAAATAAACAAGCGCAGAAGG
hsr-ϖ	Forward 20mer<br>GCAAGGGCCCACGTAGTATT	Reverse 20mer<br>AGGGGGTTTGCGTATGGTTT
pncr004:X	Forward 21mer<br>TCTGAAGAGTCCCACTTCCGA	Reverse 19mer<br>ATCATCGTCGTTGTCGCCA
reaper	Forward 20mer<br>GAGCAGAAGGAGCAGCAGAT	Reverse 20mer<br>GGACTTTCTTCCGGTCTTCG
hid	Forward 20mer<br>TCATCTTCGTCCTCCGCATC	Reverse 20mer<br>CATCCTGACCCACTCGTAGA
p53	Forward 21mer<br>CTTCAAGTTCGTCTGCCAAAA	Reverse 21mer<br>GCGCTTCTTGCTATTGAGCTG
mei-41	Forward 20mer<br>GATCGCATCTTCCGGCAAAC	Reverse 20mer<br>CTGACTCCTGGAAGCCACTG
chk-2	Forward 21mer<br>GCTGCTGATCAACCAAATGCT	Reverse 21mer<br>GTGGCTCAAGGAAGTTCTCCT
dpp	Forward 20mer<br>GGCTTCTACTCCTCGCAGTG	Reverse 20mer<br>TGCTTTTGCTAATGCTGTGC
dally	Forward 21mer<br>ACCGAAATCATGAACCATCTG	Reverse 21mer<br>CAAAGAAGTGCCTGACAGCCT
mad	Forward 20mer<br>TCCGTGATCGTTGACGGTTT	Reverse 20mer<br>GGAGTCGGATAGGCATTCGG
mei-p26	Forward 20mer<br>GCTGATCAAATCTGCGGCTG	Reverse 20mer<br>AAACACGAGAAACGTTGCCG
bam	Forward 19mer	Reverse 23mer

	GACCGAAAGCCACAAGTCG	GGACCTCCCAGTTTGTATCTCTG
nos	Forward 20mer<br>CGCGATCCTTGAAAATCTTTGC	Reverse 22mer<br>CGAACTCCTGCATCACATCCTG
pum	Forward 20mer<br>CATGTGCTTGAACACGGCAA	Reverse 20mer<br>TTGAAGGTGCAGACCTCGTC

Supplementary Table S2. Expression profiles of the differentially expressed putative lncRNAs

No	Gene	Gene ID	wild-type	*egg*[2138]	*egg*[2138]/wild-type (log2)	chromosome	range
1	CR41440	FBgn0084049	1.000	874.260	**9.772**	2R	619019 – 619416
2	CR40282	FBgn0039979	1.036	735.304	**9.471**	2R	617944 – 618746
3	CR42646	FBgn0261429	22.799	10675.424	**8.871**	2R	1250758 – 1254863
4	CR43086	FBgn0262532	4.145	1073.043	**8.016**	3L	17861840 – 17863673
5	CR31781	FBgn0051781	360.634	52884.985	**7.196**	2L	16869487 – 16878300
6	*msa*	FBgn0263610	2.073	279.840	**7.077**	3R	12656838 – 12748955
7	CR43310	FBgn0263002	2.073	178.519	**6.428**	3R	21093731 – 21094186
8	CR43087	FBgn0262533	3.109	251.856	**6.340**	3L	17867815 – 17869561
9	CR10102	FBgn0033927	264.258	16063.796	**5.926**	2R	10247938 – 10249285
10	CR43425	FBgn0263344	5.182	263.436	**5.668**	4	888450 – 890172
11	*roX2*	FBgn0019660	2.073	101.321	**5.611**	X	11473089 – 11474465
12	CR42530	FBgn0260435	14.508	643.633	**5.471**	2LHet	176023 – 179707
13	CR31451	FBgn0051451	32.125	1393.412	**5.439**	3R	19809225 – 19810787
14	CR43417	FBgn0263336	11.399	479.588	**5.395**	2R	12705024 – 12706745
15	CR43432	FBgn0263380	6.218	257.646	**5.373**	3L	14969872 – 14970726
16	CR42875	FBgn0262149	6.218	209.398	**5.074**	3R	2953809 – 2954789
17	*Cyp6a16Psi*	FBgn0031726	4.145	131.235	**4.985**	2L	5621602 – 5623760
18	*pncr003:2L*	FBgn0047133	512.971	13346.451	**4.701**	2L	16838593 – 16841887
19	*αγ-element*	FBgn0052865	155.446	3586.780	**4.528**	3R	8301058 – 8303989
20	CR43458	FBgn0263412	5.182	115.796	**4.482**	3R	26958052 – 26958772
21	*bxd*	FBgn0020556	97.413	2091.082	**4.424**	3R	12567847 – 12598911
22	CR42839	FBgn0262028	10.363	210.363	**4.343**	3R	11830043 – 11833795
23	*pncr004:X*	FBgn0047095	29.017	514.327	**4.148**	X	19065110 – 19068929
24	CR18228	FBgn0037560	145.083	2235.827	**3.946**	3R	4086428 – 4087620
25	CR41620	FBgn0085826	31.089	451.604	**3.861**	U	9844213 – 9844736
26	CR42547	FBgn0260720	8.290	118.691	**3.840**	2R	17518926 – 17521545
27	CR40743	FBgn0085772	10.363	141.850	**3.775**	U	10003811 – 10004340

28	CR40734	FBgn0085770	23.835	282.735	**3.568**	U	9836850 – 9837382
29	CR41609	FBgn0085819	100.522	1005.495	**3.322**	U	4570872 – 4571822
30	CR43614	FBgn0263589	14.508	136.060	**3.229**	X	4262819 – 4267504
31	CR40546	FBgn0085742	144.046	1268.931	**3.139**	U	5726964 – 5728907
32	CR40679	FBgn0085766	13.472	113.866	**3.079**	U	9999365 – 10000376
33	CR43344	FBgn0263049	42.489	346.423	**3.027**	2L	18455717 – 18472900
34	CR40741	FBgn0085771	26.944	218.082	**3.017**	U	8863772 – 8864332
35	CR40560	FBgn0085743	26.944	168.869	**2.648**	U	3556916 – 3559306
36	CR40469	FBgn0058469	170.990	1068.218	**2.643**	X	18880 – 19093
37	CR41619	FBgn0085825	146.119	896.454	**2.617**	U	10029292 – 10030296
38	CR34335	FBgn0085364	6568.104	39923.556	**2.604**	X	3439895 – 3440143
39	CR40766	FBgn0085773	25.908	156.325	**2.593**	U	8595904 – 8596741
40	CR43148	FBgn0262631	37.307	220.012	**2.560**	2L	21624679 – 21626190
41	CR41583	FBgn0085805	291.202	1545.876	**2.408**	U	6961350 – 6963324
42	CR43216	FBgn0262852	67.360	356.073	**2.402**	X	11481316 – 11483206
43	CR41535	FBgn0085795	236.278	1169.539	**2.307**	U	8853389 – 8854056
44	CR40639	FBgn0085758	80.832	394.671	**2.288**	U	9455772 – 9456320
45	CR40641	FBgn0085760	137.829	661.967	**2.264**	U	8006044 – 8006602
46	CR41544	FBgn0085799	58.033	277.910	**2.260**	U	9578233 – 9578772
47	CR43211	FBgn0262847	64.251	307.824	**2.260**	X	11491781 – 11493671
48	CR40668	FBgn0085764	2557.602	12214.545	**2.256**	U	9469614 – 9470353
49	CR41539	FBgn0085796	176.172	829.871	**2.236**	U	9757371 – 9757988
50	*pncr009:3L*	FBgn0062928	129.538	603.104	**2.219**	3L	19479846 – 19481488
51	CR40642	FBgn0085761	130.574	602.139	**2.205**	U	9503347 – 9503912
52	CR41613	FBgn0085822	253.895	1168.574	**2.202**	U	9112426 – 9113000
53	CR40677	FBgn0085765	43.525	198.783	**2.191**	XHet	184014 – 184944
54	CR41540	FBgn0085797	560.641	2515.667	**2.166**	U	8839005 – 8840283
55	CR41602	FBgn0085813	787.592	3343.609	**2.086**	XHet	190010 – 196035
56	CR40640	FBgn0085759	66.324	276.945	**2.062**	U	9660271 – 9660839

57	CR18166	FBgn0029526	143.010	588.630	**2.041**	X	373897 – 375842
58	CR40959	FBgn0085777	34.198	131.235	**1.940**	U	5913471 – 5914235
59	CR43361	FBgn0263093	262.185	1004.530	**1.938**	4	927816 – 928308
60	CR43213	FBgn0262849	89.122	319.404	**1.842**	X	11487595 – 11489485
61	CR40963	FBgn0085779	44.561	157.290	**1.820**	U	3653041 – 3653240
62	CR40779	FBgn0085774	605.202	2080.468	**1.781**	U	9063480 – 9064690
63	CR13656	FBgn0039307	186.535	630.123	**1.756**	3R	21094383 – 21095687
64	CR40712	FBgn0085768	129.538	437.130	**1.755**	U	9400405 – 9400910
65	*hsr-ω*	FBgn0001234	7522.542	25255.101	**1.747**	3R	17121849 – 17136428
66	CR40728	FBgn0085769	157.518	527.837	**1.745**	U	9901769 – 9902800
67	CR40621	FBgn0085757	554.423	1775.538	**1.679**	U	9411518 – 9412651
68	CR43459	FBgn0263413	60.106	192.028	**1.676**	3R	5919484 – 5925937
69	CR43217	FBgn0262853	36.271	114.831	**1.663**	X	11480261 – 11480977
70	CR41590	FBgn0085807	427.994	1272.791	**1.572**	U	7398712 – 7400705
71	CR43212	FBgn0262848	116.066	339.668	**1.549**	X	11489688 – 11491578
72	CR43215	FBgn0262851	111.921	327.124	**1.547**	X	11483409 – 11485299
73	CR41548	FBgn0085802	109.848	319.404	**1.540**	U	9795507 – 9796558
74	CR42195	FBgn0085828	73.578	208.433	**1.502**	U	4085793 – 4086544
75	CR41605	FBgn0085815	234.205	624.333	**1.415**	U	2950223 – 2951682
76	CR41607	FBgn0085817	84.977	225.802	**1.410**	U	4305171 – 4306436
77	CR43281	FBgn0262970	520.225	1376.042	**1.403**	2R	668032 – 671151
78	CR41617	FBgn0085823	412.450	1013.215	**1.297**	U	9824409 – 9824915
79	CR43481	FBgn0263492	155.446	380.197	**1.290**	3L	9373013 – 9374219
80	CR43214	FBgn0262850	137.829	331.948	**1.268**	X	11485502 – 11487392
81	CR32745	FBgn0052745	62.178	136.060	**1.130**	X	6174266 – 6174785
82	CR43650	FBgn0263659	196.898	419.760	**1.092**	2R	12716605 – 12723038
83	CR18275	FBgn0029523	116.066	243.172	**1.067**	X	371883 – 373342
84	CR31514	FBgn0051514	72.541	140.885	**0.958**	3R	5605241 – 5608415
85	CR42862	FBgn0262109	64698.624	125288.345	**0.953**	3L	224106 – 230964

86	CR43242	FBgn0262887	665.308	1181.119	**0.828**	2L	22961737 – 22963456
87	CR43334	FBgn0263039	148.192	256.681	**0.793**	3L	628435 – 643900
88	*mt:lrRNA*	FBgn0013686	434460.714	732661.370	**0.754**	dmel_ mitochondrion_ genome	12734 – 14058
89	*His-Psi:CR33867*	FBgn0053867	68.396	114.831	**0.748**	2L	21539129 – 21541127
90	CR43301	FBgn0262993	202.080	305.894	**0.598**	3R	5163945 – 5165714
91	CR12628	FBgn0042701	578.258	771.008	**0.415**	2L	22226151 – 22226771
92	CR42861	FBgn0262108	425.922	523.977	**0.299**	X	13638961 – 13639509
93	CR40190	FBgn0085732	642.509	723.725	**0.172**	2RHet	338744 – 339377
94	CR43241	FBgn0262886	426.958	472.834	**0.147**	2L	22131221 – 22132435
95	CR18854	FBgn0042174	10309.167	11359.585	**0.140**	2L	9787279 – 9790745
96	CR40354	FBgn0058354	1594.874	1691.586	**0.085**	3LHet	687420 – 688819
97	CR41501	FBgn0085786	265.294	244.137	**-0.120**	2RHet	2316691 – 2318206
98	CR42653	FBgn0261505	875.678	799.957	**-0.130**	2RHet	334447 – 335080
99	CR43651	FBgn0263660	322.291	284.665	**-0.179**	2R	5440355 – 5441330
100	CR43483	FBgn0263494	488.100	423.620	**-0.204**	3L	9378746 – 9379212
101	CR43626	FBgn0263617	132.647	109.041	**-0.283**	3L	3249373 – 3251468
102	CR31953	FBgn0051953	492.245	402.391	**-0.291**	2L	3162515 – 3163147
103	*Uhg8*	FBgn0083120	777.229	601.174	**-0.371**	3L	20446393 – 20447831
104	CR31054	FBgn0051054	341.981	263.436	**-0.376**	3R	23786492 – 23787198
105	CR31032	FBgn0051032	154.410	118.691	**-0.380**	3R	25684763 – 25685587
106	CR11700	FBgn0029856	858.061	630.123	**-0.445**	X	6176311 – 6177608
107	CR43314	FBgn0263019	149.228	102.286	**-0.545**	2L	11952200 – 11971094
108	CR31044	FBgn0051044	661.163	442.920	**-0.578**	3R	25040284 – 25045389
109	*pncr013:4*	FBgn0262731	4325.539	2832.176	**-0.611**	4	488123 – 492102
110	*His-Psi:CR31614*	FBgn0051614	128.502	82.987	**-0.631**	2L	21542989 – 21543706
111	CR31292	FBgn0051292	489.136	301.070	**-0.700**	3R	11731969 – 11732472
112	CR33294	FBgn0053294	146.119	86.847	**-0.751**	3R	12685 – 21933
113	CR43080	FBgn0262510	384.469	216.152	**-0.831**	2L	454754 – 455313
114	*His-Psi:CR33802*	FBgn0053802	172.027	96.497	**-0.834**	2L	21418852 – 21419260

115	*His-Psi:CR33811*	FBgn0053811	146.119	76.232	**-0.939**	2L	21438910 – 21439318
116	CR32010	FBgn0052010	2785.589	1440.695	**-0.951**	4	33566 – 45680
117	CR40450	FBgn0085734	250.786	122.551	**-1.033**	U	2072072 – 2074236
118	*pncr012:2L*	FBgn0259936	1613.528	732.410	**-1.139**	2L	20647533 – 20648848
119	CR32207	FBgn0052207	1361.705	607.929	**-1.163**	3L	19470429 – 19471769
120	CR32205	FBgn0052205	304.674	132.200	**-1.205**	3L	19415938 – 19417283
121	*pncr011:3L*	FBgn0083068	691.216	289.490	**-1.256**	3L	11089757 – 11091660
122	CR32011	FBgn0052011	3195.966	1308.494	**-1.288**	4	26789 – 32391
123	CR32009	FBgn0052009	2482.988	996.810	**-1.317**	4	48156 – 52259
124	*swaPsi*	FBgn0016974	1107.811	368.617	**-1.588**	X	6255528 – 6256993
125	CR18217	FBgn0036646	4276.833	1392.447	**-1.619**	3L	16593548 – 16595795
126	CR41604	FBgn0085814	2630.143	772.938	**-1.767**	U	3443350 – 3449605
127	CR34262	FBgn0085291	821.790	232.557	**-1.821**	3L	21952611 – 21953348
128	CR41597	FBgn0085810	2473.661	686.091	**-1.850**	3LHet	899 – 1989
129	CR42722	FBgn0261639	3714.119	911.893	**-2.026**	3L	24539238 – 24540086
130	CR42723	FBgn0261640	3177.313	764.253	**-2.056**	3L	24542736 – 24543545
131	CR43482	FBgn0263493	5206.399	997.775	**-2.383**	3L	9379212 – 9379563
132	CR41507	FBgn0085790	117.103	20.264	**-2.531**	YHet	312456 – 313714
133	*Uhg5*	FBgn0083123	1509.897	199.748	**-2.918**	2R	10509111 – 10512013
